# Phase-Dependency of Medial-Lateral Balance Responses to Sensory Perturbations During Walking

**DOI:** 10.3389/fspor.2019.00025

**Published:** 2019-09-27

**Authors:** Hendrik Reimann, Tyler Fettrow, David Grenet, Elizabeth D. Thompson, John J. Jeka

**Affiliations:** ^1^Department of Kinesiology and Applied Physiology, University of Delaware, Newark, DE, United States; ^2^Department of Kinesiology, Temple University, Philadelphia, PA, United States; ^3^Department of Physical Therapy, University of Delaware, Newark, DE, United States; ^4^Department of Physical Therapy, Temple University, Philadelphia, PA, United States

**Keywords:** walking, balance, motor control, virtual reality, phase dependence, galvanic vestibular stimulation (GVS)

## Abstract

The human body is mechanically unstable during walking. Maintaining upright stability requires constant regulation of muscle force by the central nervous system to push against the ground and move the body mass in the desired way. Activation of muscles in the lower body in response to sensory or mechanical perturbations during walking is usually highly phase-dependent, because the effect any specific muscle force has on the body movement depends upon the body configuration. Yet the resulting movement patterns of the upper body after the same perturbations are largely phase-independent. This is puzzling, because any change of upper-body movement must be generated by parts of the lower body pushing against the ground. How do phase-dependent muscle activation patterns along the lower body generate phase-independent movement patterns of the upper body? We hypothesize that when a sensory system detects a deviation of the body in space from a desired state that indicates the onset of a fall, the nervous system generates a functional response by pushing against the ground in any way possible with the current body configuration. This predicts that the changes in the ground reaction force patterns following a balance perturbation should be phase-independent. Here we test this hypothesis by disturbing upright balance in the frontal plane using Galvanic vestibular stimulation at three different points in the gait cycle. We measure the resulting changes in whole-body center of mass movement and the location of the center of pressure of the ground reaction force. We find that the magnitude of the initial center of pressure shift in the direction of the perceived fall is larger for perturbations late in the gait cycle, while there is no statistically significant difference in onset time. These results contradict our hypothesis by showing that even the initial CoP shift in response to a balance perturbation depends upon the phase of the gait cycle. Contrary to expectation, we also find that the whole-body balance response is not phase-independent. Both the onset time and the magnitude of the whole-body center of mass shift depend on the phase of the perturbation. We conclude that the central nervous system recruits any available mechanism to generate a functional balance response by pushing against the ground as fast as possible in response to a perturbation, but that the different mechanisms available at different phases in the gait cycle are not equally strong, leading to phase-dependent differences in the overall response.

## 1. Introduction

The control of balance during walking for humans is an important problem because the failure to maintain balance leads to falls and often injury. In the United States alone, costs from fall-related injuries amount to 20–30 billion USD annually (Burns et al., 2016). The upright human body is mechanically unstable, with a high center of mass and a relatively small base of support. Balancing this body is already a challenge during standing, requiring continuous regulation of muscle activity by the central nervous system (Morasso and Schieppati, 1999). As a result of intensive research over the last decades, we understand this neural control of balance during standing reasonably well (e.g., Winter, 1995; Peterka, 2002; Maurer et al., 2006; Kiemel et al., 2011).

Balance control during walking has been studied much less in comparison. One reason for this is that walking is highly nonlinear, with the configuration of the body changing substantially as contact with the ground is established and lost at different points during the gait cycle. This implies that at different points in the gait cycle, the central nervous system has different options available for how to generate force along the body and against the ground to affect balance. One well-studied mechanism is to shift the location of the foot placement when taking a step, which changes the pull of gravity on the body during the subsequent swing (Hof, 2008; Wang and Srinivasan, 2014; Bruijn and van Dieën, 2018; Reimann et al., 2018a). Another mechanism is to use ankle musculature to pull on the body during single stance (Hof et al., 2010; Hof and Duysens, 2018; Reimann et al., 2018b). Compared to foot placement shift, the ankle mechanism is limited in effect by the relatively small area of contact under the stance foot. Moments across the ankle joint both pull the body sideways and the foot up, so excessively large moments will result in the foot rolling over. On the other hand, the ankle mechanism has the advantage of being able to act much faster throughout single-stance and even double stance, whereas the foot placement mechanism can only be used when taking a step. For optimal benefit, there is preliminary evidence that humans flexibly coordinate these two balance mechanisms (Fettrow et al., 2018), and possibly others, to adaptively respond to different challenges of balance during walking. A recent study by Vlutters et al. (2019) shows that the (sagittal) ankle mechanisms is activated even when the ankle joint is blocked by an orthosis, indicating that it might be controlled by a dedicated reflex.

Responses to balance-related perturbations generally depend upon the phase of the gait cycle in which they are applied.Reflexes in the lower leg, probed by cutaneous electric stimulation, are highly modulated during walking, and even reverse direction (Yang and Stein, 1990; Zehr and Stein, 1999). Changes of foot placement in response to electric stimulation of the vestibular system are also highly phase-dependent (Bent et al., 2004). In contrast, responses in the upper body to these stimulations do not, or only weakly, depend on the phase of the perturbation. Logan et al. (2014) found similar results in response to visual perturbation, where the lower body response was strongly phase-dependent and the upper body response only weakly. But during normal walking, the only way to affect the lateral translational motion of the upper body is to generate a force against the ground, using muscles along the lower body. In combination, these results pose the question of how the central nervous system achieves a phase-independent response of the upper body using a phase-dependent response of the lower body.

We hypothesize that the central nervous system flexibly coordinates different balance mechanisms in the lower body in a phase-dependent way to stabilize the movement of the whole body in a phase-independent way. Here we test this hypothesis experimentally by perturbing humans walking on a treadmill with Galvanic vestibular stimulation at three different points in the gait cycle, and analyzing how the response in the whole body center of mass (CoM) and the functional force response depend upon the phase of the perturbation. We define the functional response as the displacement between the center of pressure (CoP) and the CoM. This variable is proportional to the acceleration of the CoM when assuming a single-link inverted pendulum model of the body biomechanics (Hof et al., 2005), which has proven to be reasonable approximation in a variety of cases (Kuo, 2007) and has been shown to be related to active balance control (Vlutters et al., 2016). We expect that the functional balance response to these stimuli does not depend on the phase of the perturbation. We focus our analysis to the frontal plane, which is more challenging for balance control compared to the sagittal plane (O'Connor and Kuo, 2009).

## 2. Methods

Twenty young, healthy subjects (10 female) volunteered for this study. Subjects were between 19 and 38 years old (22.2 ± 4.57), 170.7 ± 8.4 cm tall and weighed 70.5 ± 13.5 kg. Subjects provided informed verbal and written consent to participate. Subjects with self-reported history of neurological disorder or surgical procedures involving the legs, spine or head were excluded. The experimental design was approved by the Temple University Institutional Review Board (#22499). We used a statistical power analysis based on pilot data from another experiment (Reimann et al., 2018b) to determine the number of subjects required to reliably detect functionally relevant differences in the use of ankle roll and foot placement, set to 1 mms integrated CoP-CoM displacement and 5 mm foot placement change (1−β = 0.9).

### 2.1. Experimental Design

**Figure 1:**
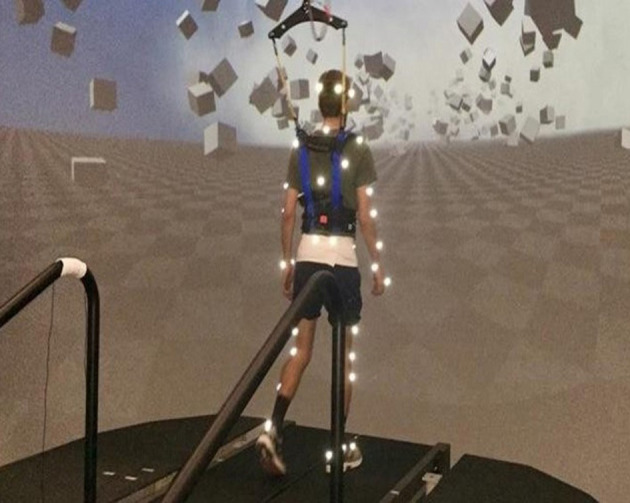
Experimental setup. Subjects walked on a self-paced treadmill immersed in a virtual environment projected onto a curved screen that covered almost their complete field of vision. The perspective in the virtual world was linked to the subject's head position.

The study was conducted in the virtual reality setup of the Coordination of Balance and Locomotion laboratory at Temple University. Subjects walked on a treadmill in a virtual environment projected onto a curved screen (Figure 1, Bertec, Inc.). This virtual reality environment is highly immersive, using a number of key components to mimic natural overground walking, listed below. The treadmill is self-paced, rather than operating at a fixed speed. This means that the treadmill belt speed dynamically adapts to the walking speed of the subject, and the subject can walk at their own pace, speeding up and slowing down as desired, without having to pay attention to their anterior-posterior position on the treadmill. The self-pacing is implemented using a nonlinear PD-controller in Labview (National instruments Inc., Austin, TX, USA) to keep the markers on the posterior superior iliac spine on the anterior-posterior mid-line of the treadmill. Walking patterns on a self-paced treadmill are generally very similar to walking on a fixed-speed treadmill (Sloot et al., 2014). The virtual world is speed-linked to the treadmill. Subjects progress through the virtual world at a rate determined by the current speed of the self-paced treadmill. This re-establishes the proper connection between walking speed and optic flow experienced in natural overground walking that is broken when walking on a normal treadmill in a fixed environment. The view in the virtual world is perspective-linked to the subject's head. We map the real-time positions of the two anterior head markers from the motion capture system (see below) to the view point in the virtual world. This generates a motion parallax effect, allowing subjects to change perspective in the virtual world and look around objects by moving the head. The projection screen is domed and covers almost the complete field of vision. When looking generally ahead, the subject is only just able to make out the borders of the dome. This removes any visual anchor to the fixed laboratory environment, immersing the subject in the speed- and perspective-linked virtual environment. In combination, these component create an immersive environment with very few external visual clues or constraints. Subjects can in principle stop paying attention to the fact that they are on a treadmill.

The virtual environment consisted of a tiled marble floor with floating cubes randomly distributed in a volume 0–10 m above the floor, 2–17 m to each side from the midline, and infinitely into the distance, forming a 4 m wide corridor for the subjects to walk through (see Figure 1), implemented in Unity3d (Unity Technologies, San Francisco, CA, USA). It was designed to have visual depth at mid-range to provide visual information about the movement of the body in space, but not at close range.

We used Galvanic vestibular stimulation (GVS) to induce the sensation of a fall to the side. Each stimulus was a square wave current with 500 μA amplitude and 600 ms duration between two round electrodes (3.2 cm diameter, Axelgaard Manufacturing 103 Co., Ltd, Fallbrook, CA, USA) fixed to the mastoid processes behind the ears. The amplitude was chosen to be large enough to induce a balance perturbation, but not so large as to substantially disrupt the gait pattern. Stimuli were triggered on heel-strikes of the right foot. Between the triggering heel-strike and the stimulus onset, we added a randomized delay of 0, 150 or 450 ms, which we will refer to as EARLY (0 ms), MID (150 ms) or LATE (450 ms) stimulus. The polarity of the stimulus was randomized to induce the sensation of falling either to the RIGHT or LEFT, where the direction of the fall sensation is toward the cathode of the electric current. A randomized wash-out period of 6–8 strides followed each stimulus. Heel-strikes were defined as downward threshold crossings of the vertical heel-marker position, where the threshold was set to 3 mm above the vertical heel-marker position of each foot during quiet standing.

Forty-five reflective markers were placed on the subject, using the Plug-in Gait marker set (Davis et al., 1991) with six additional markers on the anterior thigh, anterior tibia, and 5th metatarsal head of each foot. Marker positions were recorded at 250 Hz using an infrared motion capture system with 9 cameras (Vicon, Inc.). We collected surface electromyographical data bilaterally from five muscles along the legs and hips, but the data was corrupted due to problems with the measurement device (Delsys, Inc.), precluding analysis. Ground reaction forces and moments were collected at 1,000 Hz from both sides of the split-belt treadmill and transformed into a common coordinate frame to calculate whole-body CoP (Winter, 1990). The same speed command was sent to each belt of the treadmill, effectively treating it as single-belt.

After explaining the experiment, obtaining consent and placing markers and EMG sensors, subjects first walked for 15 min on the self-paced treadmill in the virtual environment to adapt to this experimental setup. We then stopped the treadmill and exposed subjects to the GVS while standing to familiarize themselves with the sensation, then asked them to respond to this balance perturbation “normally” while walking on the treadmill. Data collection blocks consisted of two alternating phases for *metronome* and *stimulus*. During metronome phases, lasting 30 s, subjects were provided an auditory metronome at 90 beats per minute and asked to use this as an “approximate guideline” for their pace, both during metronome and stimulus phases. During stimulus phases, lasting 120 s, the metronome was turned off, and subjects received GVS as described above. Data were collected only during stimulus phases. Each subject performed four blocks of walking, each block consisting of five metronome and five stimulus phases, always starting with metronome phases, for a total of 12.5 min per block. After each block, the treadmill was stopped and subjects were offered a break. This protocol was implemented in a custom Labview program that sent the head position and treadmill speed to the Unity computer via UDP and the stimulation currency to the stimulator.

#### Data Processing

We filled small gaps of up to 100 ms length in the kinematic data using cubic splines, then low pass filtered with a 4th order Butterworth filter at a cut-off frequency of 10 Hz. From the marker data, we calculated joint angle trajectories based on a geometric model with 15 segments (pelvis, torso, head, thighs, lower legs, feet, upper arms, forearms, hands) and 38° of freedom.We estimated the hip joint centers based upon pelvis landmarks (Tylkowski et al., 1982; Bell et al., 1990) and the knee joint centers and knee flexion rotational axes from reference movements using the symmetrical axis of rotation approach (Ehrig et al., 2007). We performed inverse kinematics by minimizing the distance between the measured and the model-determined marker positions (Lu and O'Connor, 1999). This optimization was performed first for the six pelvis degrees of freedom, which formed the root of the kinematic tree, then for the 6° of freedom at the lumbar and cervical joints, and last for each of the arms and legs separately. We estimated the body center of mass (CoM) position based on estimated segment CoM locations (Dumas et al., 2007) and the inverse kinematics and calculated CoM velocities and accelerations using numerical derivation by time. Force plate data was low pass filtered with a 4th order Butterworth filter at a cut-off frequency of 50 Hz.

We identified heelstrike events for each foot by finding minima in the vertical positions of the heel markers with inter-peak distances >250 ms and peak prominence >2 cm, and pushoff events as the first peak in the vertical velocity of the 2nd metatarsal marker with a prominence >0.35 ms^−1^ after each heelstrike. We visually inspected the result of this automatic identification and applied manual corrections in the rare cases where events were misidentified. We then partitioned the data into strides that started and ended with a right heel-strike. For each stimulus, we extracted data from the stride before and after the triggering right heel-strike, a total of two strides per trigger. The stride after the triggering heel-strike was analyzed as the stimulus data, and the stride before the trigger was used as the unperturbed reference. For each stride, we time-normalized the data for each step between consecutive heel-strikes. Strides containing missing kinematic data were excluded from further analysis. After removing these strides, an average of 43.2 ± 6.8 stimulus strides remained for each subject.

For the *whole-body response*, we analyze the velocity and acceleration of the CoM. Although these are not independent of each other, we chose to include both, because the acceleration is more directly related to the forces applied by the muscles, but the velocity is less susceptible to processing artifacts from numerical derivation. We represent the *functional response* by the displacement between CoP and CoM, which is proportional to the CoM acceleration when approximating the body by a single-link linear inverted pendulum (Hof et al., 2005). We characterized the *lower-body response* by changes of the swing foot placement relative to the stance foot at each step, and the *upper-body response* by the head and trunk roll angle. The roll angle was defined as the angle between the segment vector and the vertical in the frontal plane, where the head segment was the vector from the seventh cervical vertebra marker to the mid-point of the two markers on the back of the head, and the trunk segment was the vector from the mid-point of the two markers on the right and left posterior superior iliac spine to the seventh cervical vertebra marker. For each subject, we subtracted the mean of the unperturbed reference data from the stimulus data to estimate the response induced by the sensory perturbation. For visualization purposes, we also estimated the 95% confidence intervals for each trajectory across all repetitions, assuming no correlation from repeated measures within subjects. This estimate was only used to generate the shaded areas in the figures.

#### Estimation of Response Onset Time and Magnitude

Reliably estimating the onset time of the response is difficult due to the high amount of natural variability in walking. Our approach was to extract a short interval of data starting at the stimulus onset for each trigger, where we can assume that there is no change initially, but the response starts at some point during this interval. We approximated the time-dependency of each variable over this interval by fitting a piece-wise linear model in R (R Core Team, 2013) with two segments and a variable break-point (Muggeo, 2008), separately for each of the twenty subjects and six combinations of phase delay and stimulus direction. To estimate the response onset time, we used the location of the break-point between the two linear segments. To estimate the response magnitude, we used the absolute slope of the second linear segment. Note that this variable is the slope of a velocity response and has units of ms^−2^, but since it is the slope of a linear model fit with specific constraints, we chose to not refer to it as acceleration.

**Figure 2:**
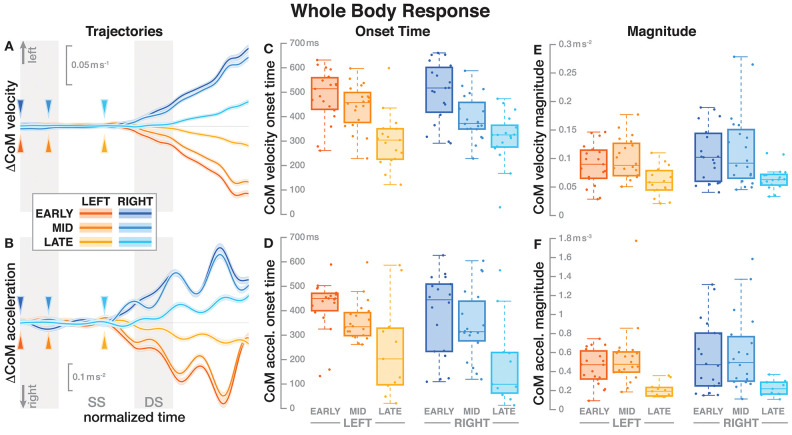
Responses in the movement pattern of the whole-body medial-lateral center of mass velocity **(A,C,E)** and acceleration **(B,D,F)**. Fall stimuli to the LEFT/RIGHT, corresponding to the side of the cathode, are shown in orange/blue tones. The left column **(A,B)** shows changes in the CoM kinematics following a perturbation. Arrows mark the approximate perturbation onset. Time is normalized, showing the two steps containing and following the perturbation, with double-stance periods shaded gray and single-stance periods white. The thick lines are average responses, the color-shaded areas are 95%-confidence intervals (see section 2). The center and right column show box plots of the estimated onset time **(C,D)** and magnitude **(E,F)** of the responses. Horizontal lines are medians, the boxes cover the first to third quartiles, whiskers the upper and lower adjacents, and the dots are single data points, where each point corresponds to one subject.

**Figure 3:**
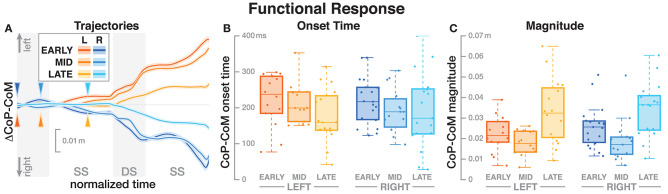
Responses in the medial-lateral center of pressure representing the hypothetical functional response to the balance perturbation. Fall stimuli to the LEFT/RIGHT, corresponding to the side of the cathode, are shown in orange/blue tones. **(A)** Shows changes in the CoP location following a perturbation. Arrows mark the approximate perturbation onset. Time is normalized, showing the two steps containing, and following the perturbation, with double-stance periods shaded gray and single-stance periods white. The thick lines are average responses, the color-shaded areas are 95%-confidence intervals (see section 2). **(B,C)** Show box plots of the estimated onset time **(B)** and magnitude **(C)** of the responses across subjects. Horizontal lines are medians, the boxes cover the first to third quartiles, whiskers the upper, and lower adjacents, and the dots are single data points, where each point corresponds to one subject.

The model fitting process was based on the following choices and assumptions. We assumed that there is no response initially, followed by a change of the outcome variable in a pre-determined direction after the onset time. This direction of change was expected to be the same as the fall stimulus direction for the CoP-CoM displacement, and the opposite for the CoM velocity and acceleration. To reflect these assumptions, we constrained the slope of the first linear segment to 0, and treated cases where the confidence interval for the break-point or slope included 0, or where the slope had the incorrect sign as missing data. The length of the analysis interval was 450 ms for the CoP-CoM displacement and 750 ms for the CoM velocity and acceleration. This value was chosen to be long enough to encompass the initial response to each stimulus, but short enough to avoid the more complex later modulations of each variable (see Figures 2, 3).

For the upper body variables head roll and trunk roll angle, the systematic response to the stimulus was so small relative to the natural variability from walking that the segmented model approach described above did not result in reliable estimates. For these two variables, we defined the response magnitude as the maximal excursion in the stimulus direction relative to the control average over four steps following each trigger. The lower body variable foot placement change is inherently discrete, so the notion of “onset time” is not meaningful. We defined the magnitude of the foot placement response as the change of the foot placement in the direction of the fall stimulus, i.e., the cathode.

#### Statistical Analysis

For each outcome variable, we performed a linear mixed effects analysis using R (R Core Team, 2013) and *lme4* (Bates et al., 2014), with fixed effects *phase delay* (EARLY, MID, LATE) and *direction* (LEFT, RIGHT), and random factor *subject*. We tested for significance of the fixed effects using an ANOVA with Satterthwaite's approximation method (Fai and Cornelius, 1996), implemented in the R-package *lmerTest* (Kuznetsova et al., 2017). We addressed multiple comparisons (ANOVAs for 10 different variables) by Bonferroni correction. Where the ANOVA reported a significant effect, we performed *post-hoc* pairwise *t*-tests with Bonferroni-adjusted *p*-values. In all tests, we used α = 0.05 as significance threshold.

We initially analyzed the effect of gender as a factor in all models. Gender had no significant effect on any outcome variable (*p* > 0.05). Comparing the model with gender to a model without gender as a factor, both the Akaike information criterion and the Bayesian information criterion were consistently lower for the model without gender. Based on these results, we used the more parsimonious models without gender as a factor for further analysis.

## 3. Results

Subjects were able to successfully complete the experimental task of walking under intermittent Galvanic stimulation. There was no instance of a subject falling or utilizing the safety harness. Subjects walked with an average velocity of 0.97 ± 0.15 m s^−1^ across the unperturbed steps. Subjects responded to the vestibular stimulation by swaying in the direction against the fall stimulus as expected (Reimann et al., 2017).

### 3.1. Whole Body Response

Figure 2 shows the average CoM velocity and acceleration trajectories for the first two steps after the triggering heelstrike. Detailed results of the ANOVAs are reported in Table 1. For both CoM velocity and acceleration, *phase delay* had a significant effect on both the onset time and the magnitude of the response. The effect of *stimulus direction* was not significant for any upper body variable, nor was there any significant interaction between *phase* and *direction*. Figures 2C,D illustrate these results. Both onset and magnitude of the response clearly depend upon *phase*, whereas the pattern for the two different stimulus directions is very similar. The *post-hoc* pair-wise *t*-tests for the onset of the response show that LATE is consistently different from EARLY and MID (*p* < 0.0017 for all comparisons), while MID and EARLY are significantly different from each other for CoM velocity (*p* = 0.0072), but not acceleration (*p* = 0.3230). For the magnitude of the response, LATE is consistently different from EARLY and MID (*p* < 0.0015 for all comparisons), but there is no significant difference between EARLY and MID (*p* = 1 for velocity, *p* = 0.9283 for acceleration).

**Table 1:** Anova results for CoM velocity and acceleration.

	**Sum Sq**	**Mean Sq**	**NumDF**	**DenDF**	***F*-value**	**Pr(>F)**
**Onset time of the CoM velocity response**						
Direction	0.00	0.00	1.00	86.57	0.10	0.7548
Delay	0.62	0.31	2.00	88.80	38.04	0.0000
Direction:delay	0.03	0.01	2.00	86.57	1.59	0.2101
**Magnitude of the CoM velocity response**						
Direction	0.00	0.00	1.00	85.58	4.06	0.0469
Delay	0.05	0.02	2.00	87.14	23.14	0.0000
Direction:delay	0.00	0.00	2.00	85.59	0.35	0.7037
**Onset time of the CoM acceleration response**						
Direction	0.02	0.02	1.00	73.49	1.47	0.2296
Delay	0.42	0.21	2.00	77.67	14.06	0.0000
Direction:delay	0.00	0.00	2.00	73.48	0.15	0.8605
**Magnitude of the CoM acceleration response**						
Direction	0.12	0.12	1.00	72.89	1.69	0.1978
Delay	1.69	0.85	2.00	77.80	12.16	0.0000
Direction:delay	0.02	0.01	2.00	72.86	0.17	0.8405

### 3.2. Functional Response

The CoP relative to the CoM shifted in the direction of the perceived fall, shown in Figure 3A. The onset time of this shift did not significantly depend upon the phase delay of the stimulus (see Table 2). Figure 3B shows the estimated onset times. Visual inspection suggests a tendency for perturbations later in the step to have shorter onset times, but this was not significant. The magnitude of the CoP-CoM shift depended significantly upon the phase delay (see Table 2). Figure 3C shows that the magnitude of the response was larger for LATE than for EARLY and MID for both stimulus directions, and this increase is statistically significant (*post-hoc* pairwise *t*-test, *p* < 0.0024 for both comparisons). The magnitude for MID is smaller from EARLY for both stimulus directions, but this difference is not statistically significant (*p* = 0.2135).

**Table 2:** Anova results for onset time and magnitude of the function response (the CoP-CoM displacement).

	**Sum Sq**	**Mean Sq**	**NumDF**	**DenDF**	***F*-value**	**Pr(>F)**
**Onset time of the functional response**						
Direction	0.00	0.00	1.00	70.04	1.04	0.3103
Delay	0.02	0.01	2.00	72.34	1.82	0.1692
Direction:delay	0.00	0.00	2.00	68.66	0.55	0.5789
**Magnitude of the functional response**						
Direction	0.00	0.00	1.00	67.79	0.91	0.3433
Delay	0.00	0.00	2.00	70.86	14.11	0.0000
Direction:delay	0.00	0.00	2.00	66.30	0.03	0.9738

### 3.3. Upper Body

**Figure 4:**
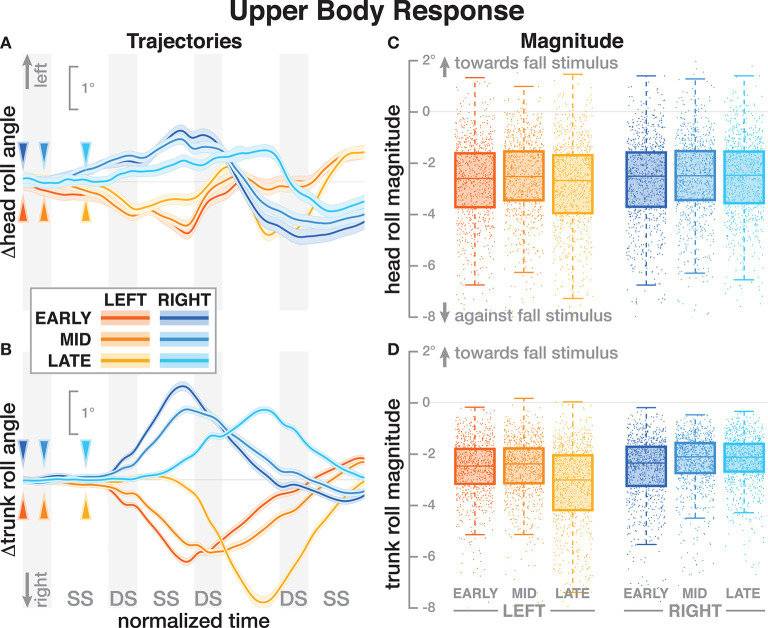
Responses in the roll angles of the head **(A,C)** and trunk **(B,D)** segments. Fall stimuli to the LEFT/RIGHT, corresponding to the side of the cathode, are shown in orange/blue tones. **(A,B)** Show changes in the roll angles following a perturbation. Arrows mark the approximate perturbation onset. Time is normalized, showing the four steps containing and following the perturbation, with double-stance periods shaded gray and single-stance periods white (note that the time shown here is twice as long as in Figures 2, 3, 5). The thick lines are average responses, the color-shaded areas are 95%-confidence intervals (see section 2). **(C,D)** Show box plots of the estimated magnitude of the responses. Horizontal lines are medians, the boxes cover the first to third quartiles, whiskers the upper and lower adjacents, and the dots are single data points, where each point corresponds to one step.

Both the head and the trunk segments leaned in the direction against the perceived fall in response to the stimulus. Figures 4A,B show the average trajectories of the head and trunk roll angle for the first four steps after the triggering heelstrike, and Figures 4C,D show box-plots for the magnitude of the response. For the head angle, the magnitude of the roll response does not depend upon the direction, nor the phase delay of the stimulus, in a statistically significant way (see Table 3). For the trunk angle, both phase delay and stimulus direction have a statistically significant effect (see Table 3). The *post-hoc* pair-wise *t*-test shows that trunk roll magnitude is significantly different between all combinations of delays (*p* < 0.0037 for all comparisons).

**Table 3:** Anova results for magnitude of the response in head and trunk roll angle.

	**Sum Sq**	**Mean Sq**	**NumDF**	**DenDF**	***F*-value**	**Pr(>F)**
**Magnitude of the head roll response**						
Direction	1.41	1.41	1.00	5150.82	0.39	0.5330
Delay	31.92	15.96	2.00	5149.00	4.39	0.0125
Direction:delay	3.66	1.83	2.00	5149.06	0.50	0.6047
**Magnitude of the trunk roll response**						
Direction	224.35	224.35	1.00	5149.76	138.60	0.0000
Delay	106.06	53.03	2.00	5148.61	32.76	0.0000
Direction:delay	338.75	169.37	2.00	5148.64	104.63	0.0000

### 3.4. Foot Placement

**Figure 5:**
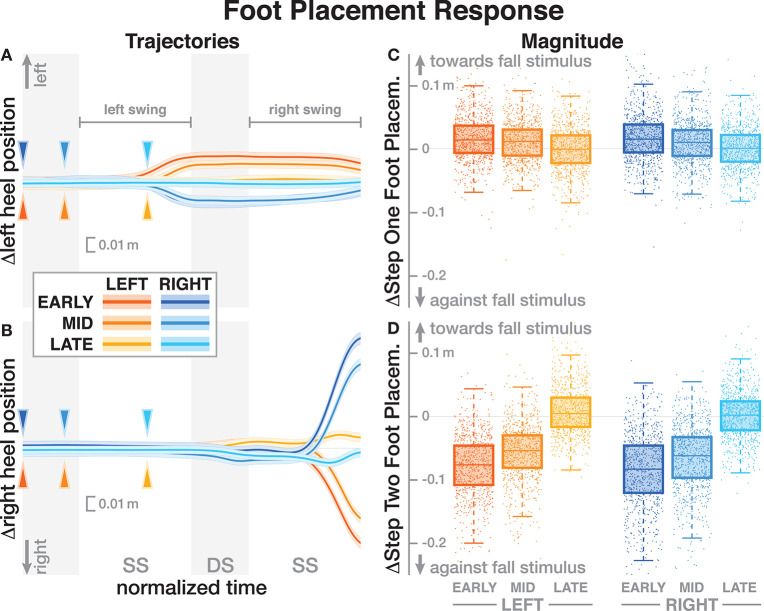
Responses of the medial-lateral foot kinematics. Fall stimuli to the LEFT/RIGHT, corresponding to the side of the cathode, are shown in orange/blue tones. **(A,B)** Show changes in the average medial-lateral heel position for both legs. Arrows mark the approximate perturbation onset. Time is normalized, showing the two steps containing, and following the perturbation, with double-stance periods shaded gray, and single-stance periods white. The thick lines are average responses, the color-shaded areas are 95%-confidence intervals (see section 2). The right column shows box plots of the estimated and magnitude of the foot placement response, i.e., the medial-lateral position of the leading foot heel relative to the trailing foot heel at each heel-strike. Step one **(C)** corresponds to the time in the middle of the trajectories on the left. Step two **(D)** corresponds to the time at the end of the trajectories on the left. Horizontal lines are medians, the boxes cover the first to third quartiles, whiskers the upper and lower adjacents, and the dots are single data points, each point corresponds to one step.

The foot placement at the fist post-stimulus step was shifted in the direction of the perceived fall, but only for the EARLY and MID phase delays (see Figure 5). At the second post-stimulus step, the foot placement was strongly shifted in the opposite direction for EARLY and MID. In the LATE condition, neither the first nor the second foot placement seems to have shifted. The left side of Figure 5 show the trajectories of both heel markers for the first two steps following the trigger, and the right side shows box plots for the magnitude of the foot placement change. Table 4 reports the results of the ANOVAs for magnitude of the foot placement change. Phase delay has a significant effect on both the first and the second step. Stimulus direction is not significant on the first step, but is significant on the second step. There is no interaction between delay and direction. The *post-hoc* pairwise *t*-tests show that foot placement is significantly different between all combinations of delays on both post-stimulus steps (*p* < 0.0001 for all comparisons).

**Table 4:** Anova results for magnitude of the foot placement response the two post-stimulus steps.

	**Sum Sq**	**Mean Sq**	**NumDF**	**DenDF**	***F*-value**	**Pr(>F)**
**Magnitude of the foot placement response in step ONE**						
Direction	0.00	0.00	1.00	5177.21	0.20	0.6551
Delay	0.24	0.12	2.00	5168.41	100.97	0.0000
Direction:delay	0.00	0.00	2.00	5168.39	1.00	0.3672
**Magnitude of the foot placement response in step TWO**						
Direction	0.08	0.08	1.00	5162.46	46.39	0.0000
Delay	7.20	3.60	2.00	5161.56	2013.28	0.0000
Direction:delay	0.00	0.00	2.00	5161.56	1.33	0.2633

## 4. Discussion

We used Galvanic vestibular stimulation to perturb the sense of balance in walking humans at three different phases in the gait cycle. We analyzed the whole-body response to this sensory perturbation in the form of changes of the center of mass (CoM) movement, and the displacement of the center of pressure (CoP) relative to the CoM, which is one aspect of the ground reaction forces generating the whole-body movement changes. Based on previous studies, we expected that the whole-body response would be phase-independent, and hypothesized that the functional response of the CoP-CoM displacement would also be phase-independent.

Our results broadly support and extend the finding that vestibular information is used to maintain balance during walking and that the balance response to vestibular stimuli depends on the phase of the gait cycle (Bent et al., 2004; Iles et al., 2007; Dakin et al., 2013). Bent et al. (2004) reported that upper-body kinematic responses to GVS were independent of the phase of the gait cycle but that the foot-placement response was phase dependent. Dakin et al. (2013) found that there is vestibular influence on motor output across many muscles throughout the gait cycle, but that the contribution of any individual muscle to the overall response is highly phase-dependent.

Subjects responded to the fall stimulus by shifting their CoP in the direction of the perceived fall, i.e., the cathode, which resulted in a body sway in the opposite direction, toward the anode, as expected. The onset time of this functional response did not depend on the phase of the perturbation (see Figure 3 and Table 2). This indicates that although different balance mechanisms are recruited at different points in the gait cycle, the neural controller tends to respond as fast as possible with any available mechanism. For the EARLY and MID perturbations, the foot placement following the perturbation was shifted in the direction of the perceived fall, but the first CoP shift occurred during single stance, indicating that the controller recruits the immediately available mechanism first and does not wait until other, potentially stronger mechanisms become available.

Contrary to our expectations and to results by Bent et al. (2004), the onset time of the whole-body response did depend on the phase of the perturbation. Responses to the LATE perturbations were consistently faster than to the EARLY and MID perturbations, with no significant difference between the latter two conditions (see Figure 2 and Table 1).

The magnitude of both the initial CoP shift in the direction of the perceived fall and the whole-body sway in the opposite direction depended upon the phase of the perturbation. The whole-body response was consistently smaller for the LATE perturbation compared to EARLY and MID (see Figure 2 and Table 1). The functional response, in contrast, was consistently larger for the LATE perturbations (see Figure 3). This is not consistent with Bent et al. (2004), who found that the size of the upper-body response did not depend on the timing of the stimulus. This may be because we used a smaller GVS stimulus than (0.5 mA vs. 1–1.5 mA, Bent et al., 2004) and also different stimulus timing, with our LATE stimulus falling in between their mid-stance and toe-off stimuli.

On the whole, there were few significant differences between EARLY and MID perturbations, but both of these were consistently significantly different from the LATE perturbations. The CoM responses were faster and smaller, whereas the CoP shift responses were larger, but with unchanged onset time. In other words, the onset time of the functional response is phase-independent, but that of the whole-body response is not, and the magnitude of both responses is phase-dependent, but in opposite directions. This difference in response pattern is peculiar, because as noted earlier, all changes of whole-body movement have to be generated by pushing against the ground using the lower body. So how can a smaller CoP shift generate a larger CoM movement change?

One possible explanation is that these differences in the whole-body movement are generated by combinations of forces that do not affect the location of the CoP, so the proportional relationship between CoP and CoM movement based on the single-link inverted pendulum assumption does not hold. One example of such forces is a hip-roll mechanism, where the upper body is rotated around the hip, generating a shear force that pushes the CoM sideways without shifting the CoP (Horak and Nashner, 1986; Reimann et al., 2018a). While this hip mechanism is usually discussed in standing or the single-stance period during walking, a biomechanical equivalent exists during double stance. This would involve differences in the trunk roll angle response early after the stimulus. Figure 4 shows that there are indeed differences, but they are neither large nor conclusive. Explaining such details satisfactorily generally requires a detailed model of the whole sensorimotor control loop, including neural dynamics, muscle physiology, biomechanics, and interaction with the environment. While such models exist for standing (e.g., van der Kooij et al., 1999; Peterka, 2000), there is no comparable model to explain balance control during walking. The available models of walking are not sufficiently detailed to explain the results presented here (Taga, 1998; Geyer and Herr, 2010; Song and Geyer, 2015).

We hypothesized that the CNS generates a functional, phase-independent motor response to balance perturbations by recruiting different balance mechanisms in a phase-dependent way. Based on this hypothesis, we predicted that the CoP shift relative to the CoM in response to a sensory fall stimulus would not depend on the phase-shift in the gait cycle in which that stimulus occurred. This prediction was partially supported by the data, in that the onset of the response was phase-independent, but also partially contradicted, in that the magnitude was phase-dependent. It is important to point out that lack of statistical significance of *phase* for the functional response is not sufficient evidence of phase-independence. It is possible that there is a phase-dependent difference, but this study was not sufficiently powered to detect it. Indeed, visual inspection of Figure 3B indicates a similar onset time pattern between LEFT and RIGHT, but more data is necessary to tell if this is an actual effect.

Our results indicate that the CNS recruits available balance mechanisms as fast as possible to respond to a sensed threat to upright stability, but the strength of the response is initially limited during single stance and only realized after a period when other mechanisms also contribute. The reason for this limitation is likely biomechanical. Responses to the EARLY and MID perturbations begin during single stance, where only the ankle roll mechanism is available. The degree to which ankle roll can shift the CoP is limited by the surface size of the foot sole (Hof et al., 2010), which is a possible explanation for the reduced magnitude of the CoP shift in EARLY and MID compared to LATE perturbations.

All stimuli were triggered on the right heel-strike, but the direction of the fall stimulus, determined by the polarity of the GVS current, was randomized. The direction of the stimulus had relatively little effect. Statistical analysis resulted in significance of *direction* after Bonferroni correction only for the magnitude of the trunk roll response. Visual inspection of the results supports this. For the whole body response (Figure 2), functional response (Figure 3) and the lower body response (Figure 5, the patterns of both onset time and magnitude of the response are very similar between LEFT and RIGHT stimuli. Only magnitude of the trunk roll angle response (Figure 4) in the LATE condition seems to be a distinct departure from this pattern. We observed a similar lack of dependence on direction in our previous study using visual perturbations (Reimann et al., 2018b). This lack of effect is slightly surprising, since one might expect that a perceived fall toward the stance foot is less threatening to overall stability than away from the stance foot.

Such a limitation of balance responses during single stance might be the underlying reason that drives some populations with balance deficits to adapt their gait patterns in characteristic ways. For example, people with Parkinson's disease tend to have shorter single-stance times than age-matched controls for similar walking speed (Morris et al., 1994). This might be because they have problems using the ankle roll mechanism for balance control and reduce the duration of the single stance period as a coping strategy.

The details of which balance mechanisms are recruited at which point in the gait cycle are still not well understood. One striking observation from our data is that for the LATE perturbation, the onset of the average CoP shift appears to align with the transition to double stance (Figure 3). But since there is no change in foot placement (Figure 5), this CoP shift cannot be generated by the well-understood foot placement mechanism. One possibility is that the transition to double stance allows recruitment of the push-off mechanism to shift weight between the two stance legs by modulating the push-off force of the trailing limb around that transition (Kim and Collins, 2015; Reimann et al., 2018b). Understanding this phenomenon requires a detailed kinematic analysis that we will perform in future work.

## Data Availability

The datasets generated for this study are available on request to the corresponding author.

## Ethics Statement

This studies involving human participants were reviewed and approved by University of Delaware IRB. The patients/participants provided their written informed consent to participate in this study. Written informed consent was obtained from the individual(s) for the publication of any potentially identifiable images or data included in this article.

## Author Contributions

HR, TF, and JJ designed the experiment. HR, TF, and ET performed the experiment. HR, TF, DG, ET, and JJ analyzed the data. HR, TF, DG, and JJ wrote the manuscript.

### Conflict of Interest Statement

The authors declare that the research was conducted in the absence of any commercial or financial relationships that could be construed as a potential conflict of interest.
